# Increased Adiposity in Adults Born Preterm and Their Children

**DOI:** 10.1371/journal.pone.0081840

**Published:** 2013-11-20

**Authors:** Sarah Mathai, José G. B. Derraik, Wayne S. Cutfield, Stuart R. Dalziel, Jane E. Harding, Janene Biggs, Craig Jefferies, Paul L. Hofman

**Affiliations:** 1 Liggins Institute, University of Auckland, Auckland, New Zealand; 2 Gravida: National Centre for Growth and Development, Auckland, New Zealand; 3 Children’s Emergency Department, Starship Children’s Hospital, Auckland District Health Board, Auckland, New Zealand; The Ohio State Unversity, United States of America

## Abstract

**Background:**

Preterm birth is associated with abnormalities in growth, body composition, and metabolism during childhood, but adult data are scarce and none exist for their offspring. We therefore aimed to examine body composition and cardiovascular risk factors in adults born preterm and their children.

**Methods:**

A cohort of 52 adults (aged 35.7 years, 54% female, 31 born preterm) and their term-born children (n=61, aged 8.0 years, 54% female, 60% from a preterm parent) were studied. Auxology and body composition (whole-body dual-energy X-ray absorptiometry) were measured, and fasting blood samples taken for metabolic and hormonal assessments.

**Results:**

Adults born preterm had greater abdominal adiposity, displaying more truncal fat (p=0.006) and higher android to gynoid fat ratio (p=0.004). Although women born preterm and at term were of similar weight and BMI, men born preterm (n=8) were on average 20 kg heavier (p=0.010) and of greater BMI (34.2 vs 28.4 kg/m^2^; p=0.021) than men born at term (n=16). Adults born preterm also displayed a less favourable lipid profile, including lower HDL-C concentrations (p=0.007) and greater total cholesterol to HDL-C ratio (p=0.047). Children of parents born preterm tended to have more body fat than the children of parents born at term (21.3 vs 17.6%; p=0.055). Even after adjustment for mean parental BMI, children of parents born preterm had altered fat distribution, with more truncal fat (p=0.048) and greater android to gynoid fat ratio (p=0.009).

**Conclusions:**

Adults born preterm, particularly men, have markedly increased fat mass and altered fat distribution. A similar increase in abdominal adiposity was observed in the term born offspring of parents born preterm, indicating that adverse outcomes associated with preterm birth may extend to the next generation.

## Introduction

Several studies have described metabolic abnormalities in subjects born preterm (<37 weeks gestation), including reduced insulin sensitivity [1-3], increased blood pressure [3-7], and a greater risk of diabetes mellitus [8]. Preterm birth also affects growth in childhood, and appears to alter the endocrine regulation of postnatal growth in childhood and adolescence [9]. Nonetheless, although children born preterm are usually shorter than children born at term [9], they experience ongoing catch-up growth during adolescence, so that final height is usually appropriate for parental height [10]. Weight gain is also initially abnormal, but unlike height, does not normalize with age. Body composition is altered from early postnatal life, with elevated fat mass and reduced lean mass by term-corrected age [11]. Importantly, fat distribution is also altered, with increased visceral and reduced subcutaneous fat compared with healthy term neonates [12]. 

Longitudinal studies have evaluated postnatal growth and weight gain in children born preterm, and related these findings to size and measures of adiposity later in adolescence or early adulthood [13,14]. However, body composition data in older survivors of preterm birth are scarce. Two studies have previously examined body composition in young adults born very preterm (≤33 weeks gestation), observing increased total body fat and greater abdominal adiposity [15,16]. 

Transmission of an environmentally acquired phenotype to subsequent generations is well described in animal studies of nutritional deprivation [17], as well as in certain human cohorts such as Dutch Famine survivors [18]. However, such inter-generational transmission has not been documented in preterm subjects. We therefore aimed to investigate body composition and cardiovascular risk factors in adults in their mid-thirties who were born preterm, and to determine whether any observed changes were also present in their offspring. 

## Materials and Methods

### Ethics approval

Ethics approval for this study was provided by the Multiregion Ethics Committee (Ministry of Health, New Zealand). Written informed consent was obtained from parents or guardians, as well as verbal or written consent from each child as was appropriate to their age.

### Participants

**Figure 1:**
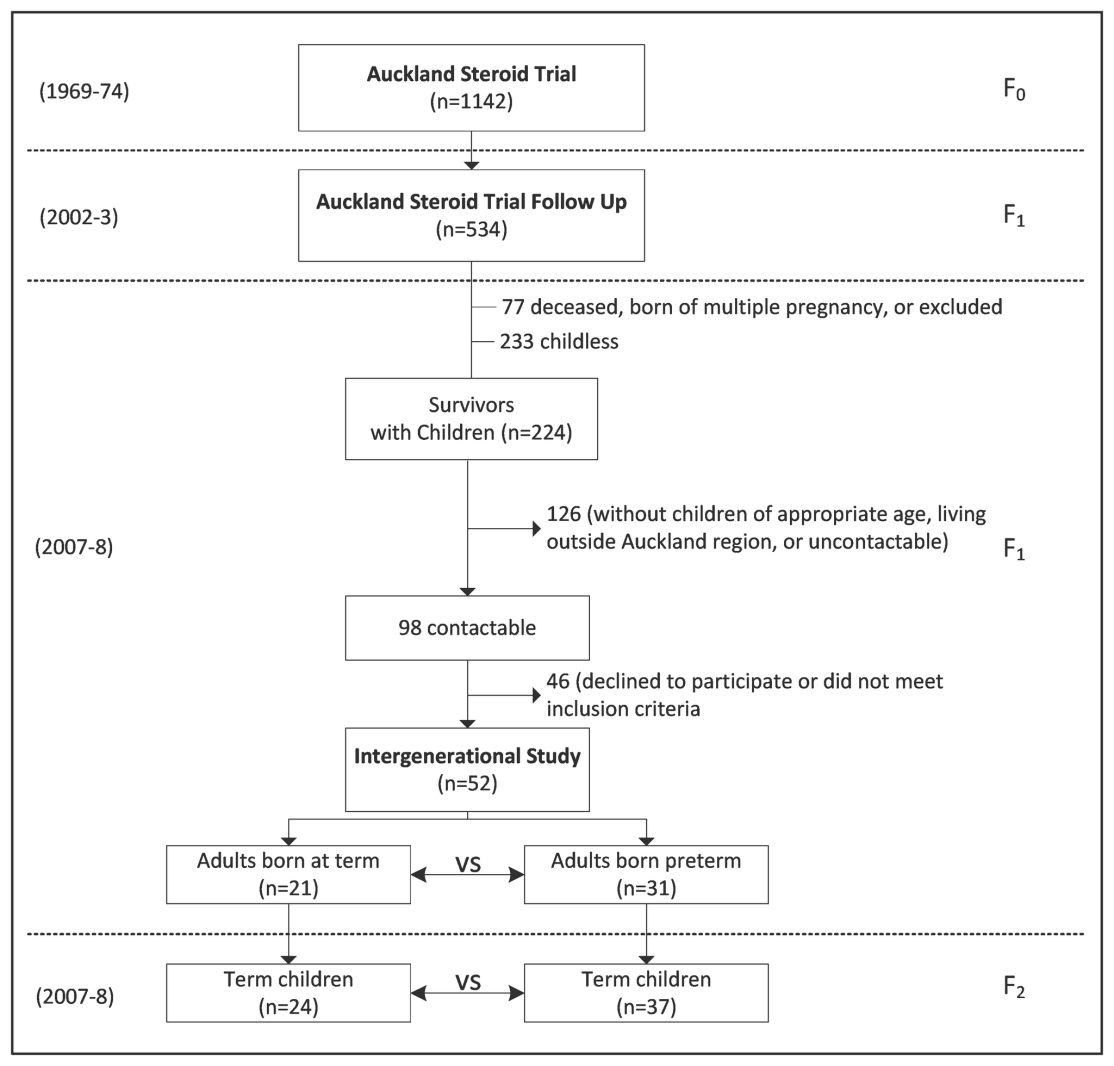
Summary of study's recruitment.

The adults for this study (F_1_) were the offspring of mothers (F_0_) from the Auckland Steroid Trial initially recruited between 1969-1974 [19] (Figure 1). This trial was the first to randomise mothers at risk of preterm delivery (defined as being <37 weeks gestation by the last menstrual period, confirmed by neonatal exam [19,20]) to receive either antenatal betamethasone or placebo (n=1142); thus, their offspring represent one of the oldest preterm cohorts in existence. Importantly, the cohort is unique in that approximately one third were born at term. Follow-up data from this cohort at a mean age of 30.6 years have previously been reported, and did not demonstrate any effects of antenatal steroid exposure on anthropometry [5,20,21].

At the time of that 30-year follow-up, details were obtained by questionnaire about subjects’ children (F_2_). We used these details to recruit all singleton adults who had singleton prepubertal children aged 5–10 years born at term (37–42 weeks gestation) (Figure 1). Adults were excluded if they were living outside the Auckland region, had chronic illness, or used medication known to affect insulin sensitivity. Children were excluded if they were born preterm, had a birth weight below the 10th percentile, had a first degree relative with diabetes, or had clinical signs of puberty (Tanner stage 2 breast development in girls and testicular volume >3 ml in boys [22,23]) or adrenarche (defined as presence of pubic or axillary hair), as these conditions may affect the outcome measures of interest. Investigators performing the studies were blinded to the perinatal characteristics of the participants.

### Clinical assessments

All children were assessed at the Maurice & Agnes Paykel Clinical Research Unit (Liggins Institute, University of Auckland). Data on each participant were collected during a single visit to the clinic. Neonatal parameters were recorded, including birth weight and gestational age. Birth weight data were transformed into standard deviation scores (SDS) [24].

Weight and height were recorded, and for the children, weight and height of the parent not involved in the original study were also recorded. Body composition was assessed using whole-body dual-energy X-ray absorptiometry (DXA) scans (Lunar Prodigy^TM^, GE Medical Systems, Wisconsin, USA). Following an overnight fast, blood samples were taken to measure plasma glucose, insulin, lipids, leptin, and cortisol concentrations. Insulin sensitivity assessments using hyperglycaemic clamps in the adults and intravenous glucose tolerance tests and Bergman’s minimal model in the children have been published previously [1]. Fasting insulin, glucose, and the derived homeostasis model assessment of insulin resistance (HOMA-IR) [25] are provided in this study. 

Physical activity levels of all participants were assessed by questionnaire, which reported weekly frequency, duration, and intensity of exercise. Levels were graded as 0 (<30 minutes at least 4 days/week), 1 (30–60 minutes at least 4 days/week), or 2 (>60 minutes at least 4 days/week). Food diaries were collected for two working days and one weekend day for each participant. Nutritional intake was estimated using standard household measures, and food labels where appropriate. Records were entered into Foodworks software (v5.0, Xyris Software, Brisbane, Australia) by a trained investigator, and the calculated mean daily caloric intake used for analysis.

### Assays

Glucose and lipids were measured on a Hitachi 902 autoanalyser (Hitachi High Technologies Corporation, Tokyo, Japan) by enzymatic colorimetric assay (Roche, Mannheim, Germany). Insulin concentrations were measured using an Abbott Imax (Abbott Laboratories, Abbott Park, USA), by microparticle enzyme immunoassay. Leptin was measured with commercially available enzyme-linked immunosorbent assays (human leptin IRMA kit DSL-10-23100, intra-assay coefficient of variation 4.4%, inter-assay coefficient of variation 4.9%). Cortisol was measured by chemiluminescence using a Roche (Indianapolis, IN, USA) E170 Modular laboratory analyser, with an inter-assay coefficient of variation of 6%.

### Statistical analyses

Potential differences between groups at baseline were tested using one-way ANOVA or non-parametric Kruskal-Wallis, while sex ratio and ethnic composition data were compared with Fisher’s exact tests (all in Minitab v.16, Pennsylvania State University, State College, PA, USA). All subsequence multivariate analyses were performed in SAS v.9.3 (SAS Institute Inc. Cary, NC, USA). 

General linear regression models and random effect mixed models were used to assess differences between groups. Important confounding factors were adjusted for in the analyses, including ethnicity, F_0_ steroid exposure, age, and gender. For adult data (F_1_) small-for-gestational-age status was also controlled for in the model, while for the offspring (F_2_) birth weight SDS was included as a covariate. Other factors were controlled for as required, depending on the outcome response of interest: for lipids, hormones, and outcomes associated with glucose homeostasis – BMI was included; and for anthropometric data – the appropriate parental factor (e.g. mean parental BMI). The interaction effects between group and gender were tested in all models, and outcomes were assessed separately for males and females if there was indication of a differential response between genders.

All statistical tests were two-tailed and maintained at a 5% significance level. Age data are presented as means ± standard deviations. Outcome data are presented as model-adjusted means (estimated marginal means adjusted for the confounding factors in the models), with associated 95% confidence intervals. Overweight and obesity for adults were defined as BMI 25–30 and ≥30 kg/m^2^, respectively.

## Results

### Adult (F_1_)

Of the 534 adult (F_1_) survivors previously traced at 30 years of age, 461 were born from singleton pregnancies, 207 had children aged 5–10 years, and 127 were living in the Auckland region (Figure 1). Of this group, 98 were contactable, 19 were excluded as a result of chronic illness, and 27 declined to participate (Figure 1). Thus, we studied 52 adults aged 35.7 ± 1.2 years (range 33.4–38.0 years) who met the inclusion criteria and agreed to participate. 31 participants were born preterm (Figure 1) with a mean gestational age of 33.3 weeks (Table 1), including 8 participants born <32 weeks gestation and the remaining 23 born 32–36 weeks. Adults born preterm and at term were of similar birth weight SDS, age, sex ratio, and ethnic composition (Table 1). 

**Table 1:** Baseline characteristics of adults (F_1_) and their children (F_2_).

	**Adults (F_1_)**		**Offspring (F_2_)**	
	**Preterm**	**Term**	**Preterm parent**	**Term parents**
**n**	31	21	37	24
**F_0_ exposure to antenatal steroids** (n)	13	10	19	11
**Gestational age** (weeks)	33.3 ± 2.2	39.7 ± 1.2****	39.7 ± 0.8	40.2 ± 0.7*
**Birth weight SDS**	-0.24 ± 1.11	-0.67 ± 0.92	-0.19 ± 1.22	-0.24 ± 0.98
**Age** (years)	35.7 ± 1.3	35.7 ± 0.9	7.9 ± 1.6	8.2 ± 1.7
**Sex ratio** (males)	52%	38%	46%	63%
**Ethnicity** (New Zealand European)	61%	67%	38%	58%

*p<0.01, ****p<0.0001 for Term vs Preterm. Where appropriate, data are means ± standard deviations.

**Figure 2:**
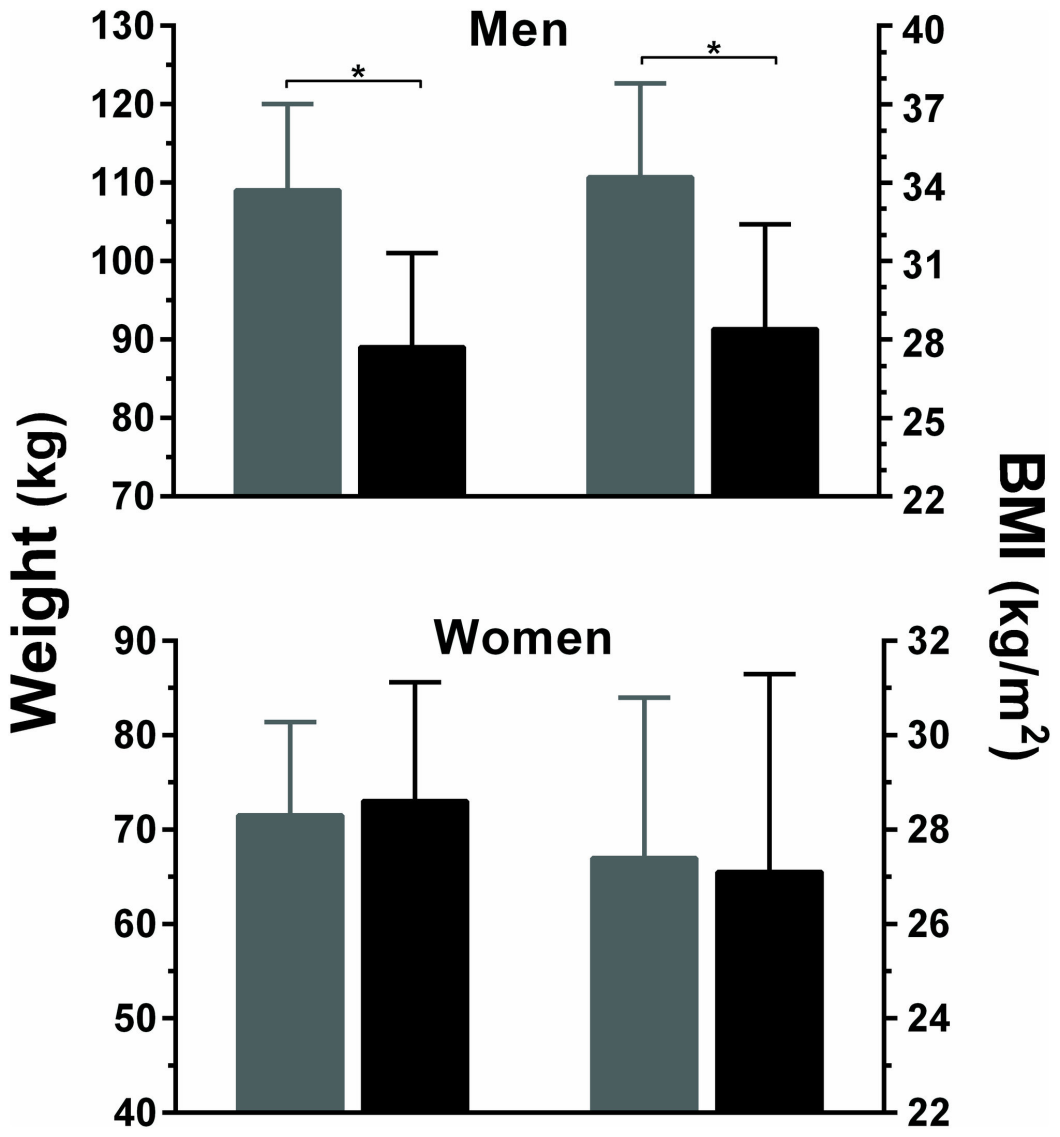
Weight and BMI in men and women (F_1_) who were born preterm (gray) or at term (black). Data are means and 95% confidence intervals adjusted for other confounding factors in the multivariate models.

Overall, there were no significant differences in height, weight, and BMI between adults born preterm and at term (Table 2). However, while women born preterm and at term were of similar weight and BMI, men born preterm (n=8) were considerably heavier (109 vs 89 kg; p=0.010) and of greater BMI (34.2 vs 28.4 kg/m^2^; p=0.021) than men born at term (n=16) (Figure 2). Overall, 21/31 (68%) of adults born preterm were overweight or obese compared to 11/21 (52%) of those born at term (p=0.27). However, 39% (12/31) of adults born preterm were obese, compared to 14% (3/21) of adults born at term (p=0.049). 

**Table 2:** Anthropometric and metabolic outcomes among adults (F_1_) born preterm or at term.

		**Adults born preterm**	**Adults born at term**	**p-value**
**n**		31	21	
**Anthropometry**	Height (cm)	170.0 (167.1–172.9)	171.5 (167.9–175.1)	0.44
	Weight (kg) **^*†*^**	88.0 (81.2–95.4)	82.9 (75.1–91.7)	0.28
	BMI (kg/m^2^) **^*†*^**	30.5 (28.3–32.9)	28.3 (25.8–31.0)	0.14
	Total body fat (%)	35.4 (32.0–38.8)	29.4 (25.2–33.6)	**0.011**
	Truncal fat (%)	38.3 (34.1–42.5)	30.1 (25.0–35.3)	**0.006**
	Android fat to gynoid fat ratio	1.09 (1.01–1.16)	0.93 (0.83–1.02)	**0.004**
**Lipid profile**	Total cholesterol (mmol/l)	4.39 (3.97–4.82)	4.65 (4.16–5.13)	0.36
	HDL-C (mmol/l)	1.12 (0.98–1.26)	1.38 (1.22–1.54)	**0.007**
	LDL-C (mmol/l)	2.88 (2.48–3.28)	3.04 (2.59–3.50)	0.53
	Total cholesterol to HDL-C ratio	4.08 (3.58–4.65)	3.40 (2.89–3.98 )	**0.047**
**Hormones & glucose homeostasis**	HOMA-IR	1.37 (1.07–1.74)	1.06 (0.80–1.41)	0.11
	Fasting insulin (mIU/l)	6.51 (5.18–8.18)	5.09 (3.91–6.62)	0.10
	Fasting glucose (mg/dl)	4.74 (4.57–4.91)	4.72 (4.52–4.92)	0.87
	Leptin (ng/ml)	2.40 (2.08–2.72)	1.98 (1.61–2.35)	**0.042**

Data are means and 95% confidence intervals adjusted for other confounding factors in the multivariate models. **^*†*^**parameters with a sex-dependant response.

Both men and women born preterm had more body fat than those born at term (p=0.011; Table 2). They also displayed altered distribution of adipose tissue with increased abdominal adiposity, namely more truncal fat (p=0.006) and higher android to gynoid fat ratio (p=0.004) than adults born at term (Table 2). 

Consistent with their greater adiposity, adults born preterm had leptin concentrations that were 21% higher than those born at term (p=0.042; Table 2). Adults born preterm also displayed a less favourable lipid profile, including lower HDL-C concentrations (p=0.007) and greater total cholesterol to HDL-C ratio (p=0.047; Table 2). 

### Children (F_2_)

We studied 61 children aged 8.0 ± 1.6 years (range 5.2–10.6 years), of whom 37 had a parent born preterm (Figure 1; Table 1). Children of parents born preterm and at term were of similar birth weight SDS, age, sex ratio, and ethnic composition (Table 1). Interestingly, children of parents born preterm were born on average 0.5 weeks earlier than children of parents born at term (p=0.039; Table 1), even though children born preterm had been excluded from this study.

Children of parents born preterm were of similar height SDS and BMI SDS as the offspring of parents born at term (Table 3), but children of preterm parents tended to have more body fat (21.3 vs 17.6%; p=0.055). Further, even after adjustment for mean parental BMI, children of preterm parents displayed altered fat distribution, with greater truncal fat (p=0.048) and android fat to gynoid fat ratio (p=0.009; Table 3). 

**Table 3:** Offspring (F_2_) of parents who were born preterm or at term.

		**Children of parent born preterm**	**Children of parents born at term**	**p-value**
**n**		37	24	
**Anthropometry**	Height SDS	0.51 (0.01–1.01)	0.54 (0.02–1.07)	0.90
	BMI SDS	0.26 (-0.22–0.75)	0.38 (-0.13–0.90)	0.65
	Total body fat (%)	19.3 (16.3–22.8)	17.2 (14.3–20.7)	0.22
	Truncal fat (%)	15.8 (13.6–18.4)	12.3 (10.1–15.1)	**0.048**
	Android fat to gynoid fat ratio	0.71 (0.63–0.81)	0.60 (0.52–0.68)	**0.009**
**Lipid profile**	Total cholesterol (mmol/l)	3.87 (3.47–4.28)	3.87 (3.45–4.28)	0.98
	HDL-C (mmol/l)	1.29 (1.13–1.44)	1.37 (1.21–1.54)	0.35
	LDL-C (mmol/l)	2.41 (2.07–2.75)	2.30 (1.95–2.65)	0.55
	Total cholesterol to HDL-C ratio	3.00 (2.71–3.29)	2.83 (2.52–3.15)	0.35
**Hormones & glucose homeostasis**	HOMA-IR	1.02 (0.66–1.37)	1.31 (0.93–1.69)	0.13
	Fasting insulin (mIU/l)	4.62 (3.18–6.06)	5.86 (4.32–7.41)	0.12
	Fasting glucose (mg/dl)	4.74 (4.49–5.00)	4.89 (4.62–5.16)	0.33
	Baseline cortisol (nmol/l)	220 (177–273)	173 (137–219)	**0.048**
	Leptin (ng/ml)	4.52 (3.25–6.31)	4.11 (2.89–5.84)	0.61

Data are means and 95% confidence intervals adjusted for other confounding factors in the multivariate models.

Baseline cortisol concentrations were 27% higher in children of parents born preterm (p=0.048; Table 3). However, leptin concentrations, lipid profiles, and parameters of glucose homeostasis were not different between groups (Table 3). Physical activity levels and mean caloric intake were similar in both groups (data not shown), and the gender of the parent born preterm did not affect auxological parameters in the offspring. 

## Discussion

This study shows that adults born preterm, particularly men, have a marked increase in adiposity (mostly abdominal/truncal) in mid-adult life. Further, the impact of preterm birth on adiposity appears to extend to the following generation, as children of parents born preterm displayed similar alterations in fat distribution, with greater central adiposity.

Our study is consistent with previous longitudinal data in young adults indicating greater adult adiposity in those born preterm [15,16]. Those studies showed subtle differences in body composition in young adults born preterm. Thus, it appears that total and abdominal adiposity are amplified with increasing age, so that in our cohort nearly half of adults born preterm were obese in mid-adulthood.

The increase in adiposity in men born preterm was predominantly due to abdominal fat, and it was associated with lower HDL-C concentrations. Further, our recent analysis of insulin sensitivity in this cohort also showed that adults born preterm had higher fasting insulin concentrations and reduced insulin sensitivity [1]. These features combined are hallmarks of the metabolic syndrome, characterized primarily by marked abdominal/visceral adiposity, fasting hyperglycaemia, hypertension, and dyslipidemia [26]. 

As in other at-risk groups (such as those born small-for-gestational-age), men born preterm appear more susceptible than women to developing this phenotype as shown by the observed differences in weight and BMI. This sexual dimorphism is consistent with data from maternal undernutrition studies [27,28]. A study in the Philippines for example, showed an inverse association between maternal protein intake during pregnancy and blood pressure in male but not female offspring in adolescence [29]. While the underlying mechanisms are still poorly understood, the placenta has been recently suggested to play a key role [30]. Oestrogen may also have an important role, and one study suggested that this hormone was a primary factor normalizing blood pressure in adult female rats that were growth-restricted *in utero* [31]. In our study, it is possible that this male susceptibility to the metabolic syndrome may be associated with a greater capacity of males to store fat and maintain body weight in response to the adverse environment associated with preterm birth (both *in utero* and post-natally). Nonetheless, although the mechanisms underpinning worse outcomes among those born preterm are yet to be determined, our study shows that preterm birth is associated with adverse changes to body composition and metabolic parameters in adulthood. 

Importantly, we also showed that preterm birth is associated with an adverse phenotype in the next generation. The offspring of parents born preterm tended to have greater fat mass than those of parents born at term, reflecting the effect of parental obesity. Fatter parents tended to have fatter children irrespective of whether the preterm parent studied was male or female. However, even when mean parental BMI was taken into account, alterations in fat distribution were still evident amongst children of parents born preterm, suggesting that parental fat mass did not entirely explain the effect of parental preterm birth on offspring adiposity.

Intergenerational effects have been observed in animal models [17], particularly in the development of diabetes [32]. Such effects also occur in humans, and prenatal exposure to the Dutch Famine led to transgenerational effects on neonatal adiposity and health in later life [33]. More recently, a Finnish study on nearly 5,000 children provided strong evidence for the intergenerational transmission of obesity [34]. The mechanisms behind this intergenerational transmission of an obese phenotype to the offspring of parents born preterm are unclear, but environmental effects leading to heritable epigenetic changes (and consequent alterations in gene expression) are well described [27,35].

Preterm birth is not uncommon, and more than 10% of babies worldwide are born less than 37 weeks gestation [36]. Although metabolic and growth abnormalities have been consistently demonstrated in individuals born less than 32 weeks gestation [2,37], these babies comprise only 1.5% of all births. It was unclear whether similar abnormalities occurred in most preterm babies, i.e. those born with a lesser degree of prematurity (32–37 weeks gestation). In our study only 25% of the preterm adults were born before 32 weeks gestation, and our findings suggest that all preterm survivors (i.e. >10% of all births) may suffer long-term adverse health effects.

The intergenerational effect of preterm birth is well established, and mothers who were born preterm are also more likely to have preterm deliveries [38]. Although fathers seem to contribute to this risk as well, the effect appears to be smaller [39]. In the current study, we showed that both maternal and paternal preterm births result in an earlier age of delivery, even though births <37 weeks gestation had been excluded. It therefore appears that earlier birth could be an inherited trait, one that is epigenetically modified, or a combination of both. Unfortunately, no follow-up data are available for more than two generations, making further conclusions difficult. However, given the changes in the metabolic status of adults born preterm as they age and the body composition alterations observed in their offspring, it is tempting to speculate that both maternal and paternal epigenetic changes may be altering gestation in future generations.

In conclusion, preterm birth is associated with increased fat mass and altered fat distribution in adulthood, particularly among men, as well as evidence of insulin resistance and less favourable lipid profiles. Importantly, similar changes in body composition are also present in mid-childhood in their offspring born at term, showing that the negative consequences of preterm birth may extend to the subsequent generation. The changes in body composition observed in this group suggest ongoing longitudinal studies will be necessary to define the health relevance of preterm birth for future generations. 
